# The Preparation and Contact Drying Performance of Encapsulated Microspherical Composite Sorbents Based on Fly Ash Cenospheres

**DOI:** 10.3390/molecules29102391

**Published:** 2024-05-19

**Authors:** Elena V. Fomenko, Natalia N. Anshits, Leonid A. Solovyov, Vasily F. Shabanov, Alexander G. Anshits

**Affiliations:** 1 Institute of Chemistry and Chemical Technology, Federal Research Center “Krasnoyarsk Science Center of the Siberian Branch of the Russian Academy of Sciences”, Akademgorodok 50/24, 660036 Krasnoyarsk, Russia; anshitsnn@mail.ru (N.N.A.); leosol@icct.ru (L.A.S.); anshits@icct.ru (A.G.A.); 2 Federal Research Center “Krasnoyarsk Science Center of the Siberian Branch of the Russian Academy of Sciences”, Akademgorodok 50, 660036 Krasnoyarsk, Russia; shabanov@ksc.krasn.ru

**Keywords:** novel materials, dehydration process, sorption, desiccant, crystalline hydrate, cenospheres, porous matrix, composite sorbent

## Abstract

Sorption technologies are essential for various industries because they provide product quality and process efficiency. New encapsulated microspherical composite sorbents have been developed for resource-saving contact drying of thermolabile materials, particularly grain and seeds of crops. Magnesium sulfate, known for its high water capacity, fast sorption kinetics, and easy regeneration, was used as an active moisture sorption component. To localize the active component, porous carriers with an accessible internal volume and a perforated glass–crystalline shell were used. These carriers were created by acid etching of cenospheres with different structures isolated from fly ash. The amount of magnesium sulfate included in the internal volume of the microspherical carrier was 38 wt % for cenospheres with ring structures and 26 wt % for cenospheres with network structures. Studies of the moisture sorption properties of composite sorbents on wheat seeds have shown that after 4 h of contact drying the moisture content of wheat decreases from 22.5 to 14.9–15.5 wt %. Wheat seed germination after sorption drying was 95 ± 2%. The advantage of composite sorbents is the encapsulation of the desiccant in the inner volume of perforated cenospheres, which prevents its entrainment and contamination and provides easy separation and stable sorption capacity in several cycles.

## 1. Introduction

Adsorption is a fundamentally efficient process for sustainable industrial development. Sorption technologies are a key separation tool in various industries—chemical, biological, pharmaceutical, energy, etc.—providing the quality of raw materials, products, and the productivity of processes in general [1,2,3]. To date, the main applications of adsorbents are separation of gas and liquid mixtures [4,5], catalysis and ion exchange [3], heat conversion/storage/amplification [6,7,8], water harvesting from atmospheric air [9], recuperation of heat and moisture in ventilation systems [10], active heat insulation [11], and air conditioning [12]. Such diverse applications often require materials with adsorption properties that can differ considerably for different applications.

Traditionally, the selection of adsorbents for a given application involves a limited range of existing materials. The most common are materials with developed surfaces, such as porous carbons [13], zeolites [14,15], aluminum oxides [15], silica gels [16,17], and metal–organic frameworks (MOFs) [18]. Some traditionally used adsorbents have a high regeneration temperature, low mechanical strength, high cost, and sometimes limited sorption capacity, which are disadvantages. For example, silica gel still has limited sorption capacity, zeolites require high regeneration temperatures, and MOFs are very expensive to manufacture.

Another, fundamentally different approach consists of the purposeful synthesis of materials with specified properties that best meet the requirements of a particular application. For composite systems, there is an additional possibility of varying the properties due to the combination of sorption-active substances and a matrix of a specific nature. The two-component composites “salt in the porous matrix” have been given deserved recognition as promising representatives of this class of materials [19,20,21,22,23]. From the first studies on the manufacture of such sorbents by impregnating a porous matrix with hygroscopic salts [19,20], numerous combinations of salts and porous carriers with enhanced sorption capacity have been developed. Active components include calcium and lithium chlorides, lithium bromide, magnesium and sodium sulfates; porous matrices include silica gels, aluminum oxide, synthetic carbon materials, vermiculite, and zeolites [24,25,26,27,28,29]. As proved, this is an effective way to create innovative materials with specified sorption properties.

Hollow micro-/nanostructures are of great interest in many areas of modern knowledge-intensive areas [30,31], including attracting attention in the search for efficient sorbents because they have a large inner space for localizing active components [32]. Encapsulated sorbents represent a new level of composite materials with enhanced physicochemical properties and performance characteristics. Similarly to two-component salt-in-porous-matrix composites [19,20,21,22,23,24,25,26,27,28,29], Yang et al. [32] developed a solid sorbent obtained by encapsulating the hygroscopic salt of lithium chloride (LiCl) inside microsized hollow-structured SiO_2_, which demonstrated a record high water vapor sorption capacity.

Hollow aluminosilicate microspheres, called cenospheres, can serve as promising carriers of active components of composite sorbents. Cenospheres are one of the valuable microspherical components of fly ashes of the aluminosilicate type (Si-Al) formed during pulverized coal combustion in TPPs, with a low bulk density of 0.2–0.8 g/cm^3^ and easily isolated in the form of concentrates by gravitational methods in aqueous media [33,34,35]. Since the cenosphere concentrates have variable compositions and consequently unstable properties, it is impossible to use them directly as functional materials, including sorbents and carriers. Currently, methods have been developed to separate narrow fractions of microspherical components with a specified composition from concentrates of all industrial fly ashes [36,37,38]. The well-characterized fractions of cenospheres with a narrow particle size distribution of a specific composition and structure are successfully used in the development of new functional materials with controlled properties. In particular, microspherical carriers [39], composite sorbents for the purification of liquid waste from radionuclides and nonferrous and heavy metal ions [39,40,41], and magnetically controlled encapsulated pH-sensitive spin probes for the study of biological objects [42] were obtained based on perforated cenospheres. Due to the presence of an inner cavity, the high strength of the glass–crystalline shell, thermostability, and acid resistance, cenospheres act as micro-containers to localize the active component in the inner volume of the carrier. This type of microspherical sorbent prevents entrainment of the dispersed sorption-active component and minimizes losses as a result of its placement in the internal cavity of the globules.

One of the promising applications of encapsulated composite sorbents is energy-saving technologies for contact drying of thermolabile materials, namely, seeds of crops, which do not tolerate thermal effects or else lose valuable properties when heated. The moisture content in seeds should not exceed a critical value that characterizes only loosely bound water, which for primary grain crops is 14.5–15.5% [43]. Drying is the main method of post-harvest processing and preservation of grain quality by removing moisture to the required level. Significant disadvantages of heat treatment are high energy consumption [44] and loss of seed germination [43,45]. Silica gel [46], clay minerals [47], and zeolites [48] have been considered as desiccants for contact drying of various crops. Due to the high dispersion ability of some reagents, there was entrainment and adherence to the grain surface, which adversely affected the process efficiency. Using composite sorbents, in which the desiccant component is in the inner volume of the carrier, will avoid these disadvantages.

In the development of encapsulated composite sorbents for contact drying of seeds, the correct choice of desiccant and porous carrier as the matrix for its localization is essential. An ideal composite sorbent should have a high water capacity, fast sorption and desorption kinetics, easy regeneration, and stability of cyclic characteristics. The efficiency of contact sorption drying directly depends on the physicochemical properties of the desiccant used. Magnesium sulfate is one of the best desiccants; its advantages include neutrality, high water sorption rate, high water capacity, and low regeneration temperature [49]. Additionally, magnesium sulfate is a well-established, inexpensive, and widely used agricultural fertilizer that does not pollute the soil and can significantly increase crop yields [50,51].

The physicochemical properties of the carrier should contribute to the practicality and efficiency of using the desiccant. Microspherical carriers with a perforated shell of specific thickness and porosity have clear advantages. As a result of the spherical shape of the carrier, it is possible to avoid mechanical damage to the grain. The presence of the inner cavity of the spherical matrix will encapsulate the active component, thus minimizing its loss, preventing adherence to the grain surface, and providing easy separation at the end of the process. The open system of transport pores associated with the surface will allow the components to contact the grain without significant diffusion limitations. Perforated cenospheres, successfully tested in the development of microspherical carriers and encapsulated sorbents [39,40,41,42], can be used as desiccant carriers for composite materials for grain contact drying.

This study aimed to develop encapsulated composite sorbents based on perforated fly ash cenospheres and magnesium sulfate for contact drying of thermolabile materials, in particular seeds of crops, and to investigate their moisture sorption properties using as an example the most important cereal crop—wheat. The research will demonstrate that the contact drying process achieves the required values of wheat moisture content while preserving seed germination. The advantage of composite sorbents is the localization of desiccant in the inner volume of microspherical carriers, which prevents its entrainment, contamination, and adhesion to the grain surface and provides stable sorption capacity at a high level in several drying cycles.

## 2. Results and Discussion

### 2.1. Preparation and Characterization of Encapsulated Composite Sorbents

**Figure 1:**
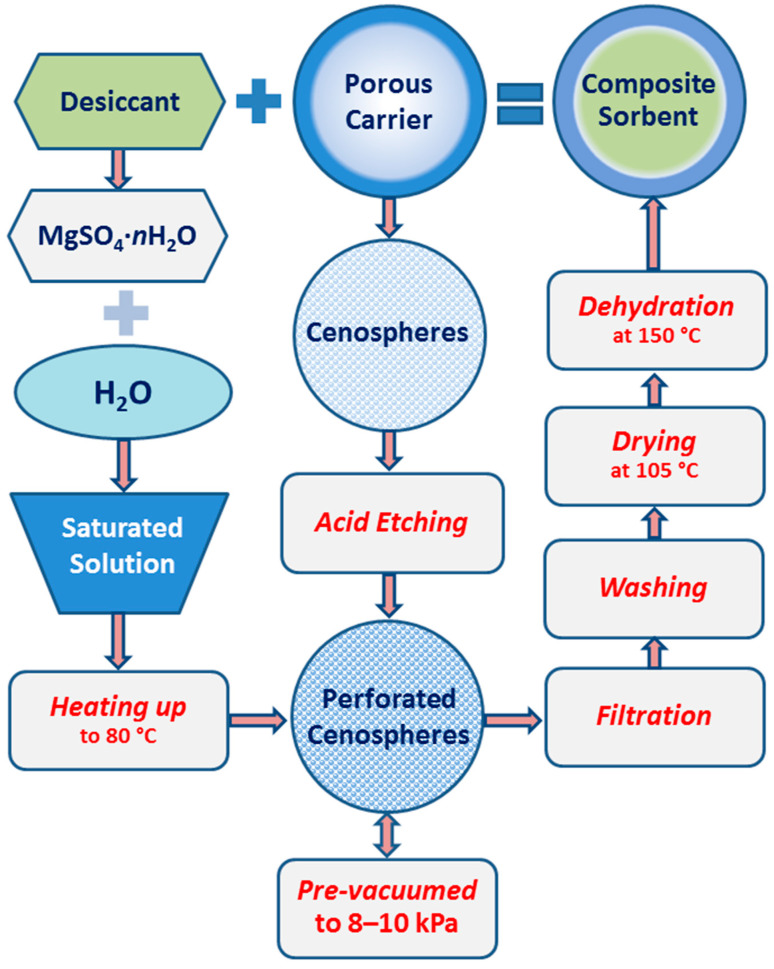
Schematic representation of the preparation of encapsulated composite sorbents.

The primary materials, magnesium sulfate and fly ash cenospheres, proposed as desiccant and carriers for the synthesis of encapsulated composite sorbents, were characterized. The main stages of the preparation process are presented schematically in Figure 1 and listed below:

- A saturated solution of magnesium sulfate is prepared by mixing the right amount of salt MgSO_4_·7H_2_O and distilled water while heating to 80 °C.
- Microspherical porous carriers are prepared by acid etching of narrow fractions of cenospheres.
- Magnesium sulfate is localized in the internal volume of microspherical carriers by precipitation from a saturated solution.
- Before filling with a saturated solution, the cenosphere cavities are pre-vacuumed through a perforated glass–crystalline shell to a residual pressure of 8–10 kPa.
- After the cenospheres are fully filled, the excess liquid phase is filtered off and the outer surface of the globules is washed with distilled water to remove excess magnesium sulfate.
- The composite sorbent is dried at a temperature of 105 °C for 90 min and dehydrated at 150 °C to a constant weight.

#### 2.1.1. Desiccant Characterization

A dehydrated chemical reagent, magnesium sulfate 7-hydrate of the “pure” grade, was used as an active moisture sorption component of microspherical composite sorbents. According to the certificate [52], the chemical reagent meets specific standards, including the mass fraction of MgSO_4_·7H_2_O—not less than 99.0; water-insoluble substances—not more than 0.002; calcium—not more than 0.02; and iron—not more than 0.0005 wt %.

In this study, the physicochemical characteristics of MgSO_4_∙7H_2_O were determined using scanning electron microscopy with energy dispersive spectroscopy (SEM-EDS), X-ray diffraction (XRD), and thermal (DSC-TG) analyses for the −0.2 mm fraction. Characterization techniques are described with details in Section 3.2.

**Figure 2:**
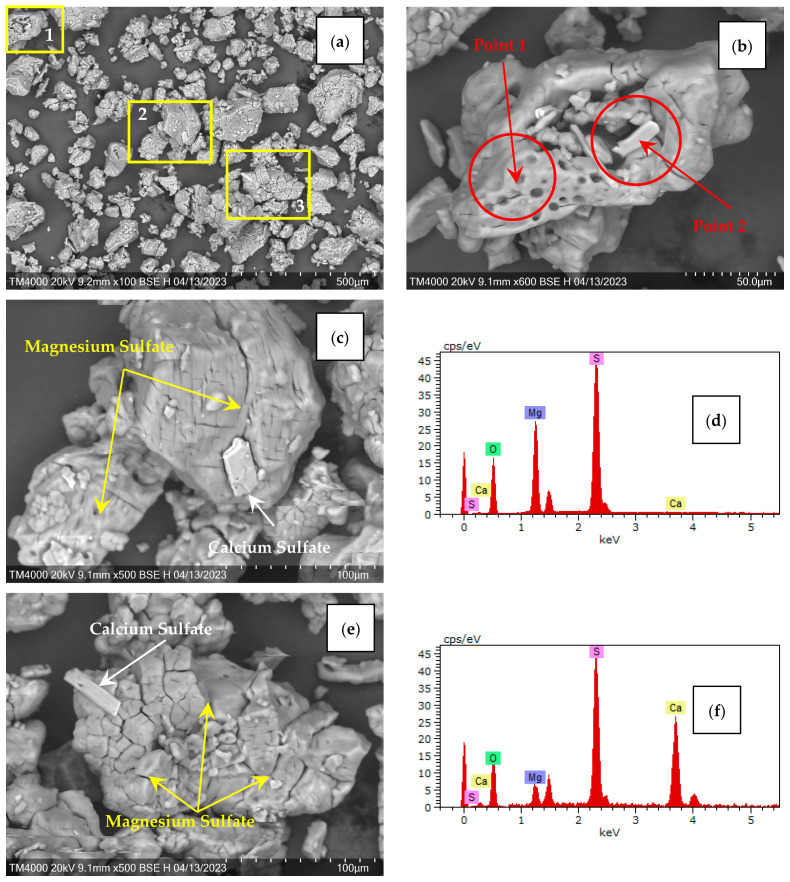
SEM images of the magnesium sulfate MgSO_4_**·***n*H_2_O −0.2 mm fraction: (**a**) General view; (**b**) Enlarged fragment 1; (**c**) Enlarged fragment 2; (**e**) Enlarged fragment 3. Energy dispersive spectra of the granule in (**b**): (**d**) Point 1; (**f**) Point 2.

Study of the morphology and chemical composition of the solid desiccant was carried out using the SEM-EDS method. This study of magnesium sulfate showed that the sample is represented by compact fragmented particles (Figure 2a) with a dense network of cracks outlined along the crystalline separation (Figure 2c,e). Flat blocky calcium sulfate crystals are ingrown up to 50 µm long on the surface of magnesium sulfate particles (Figure 2b,c,e). Figure 2d,f present the energy dispersive spectra of the local regions, indicated in Figure 2b by red circles in the absence of crystal (Point 1) and with calcium sulfate crystal (Point 2), respectively. From the data analysis, it was concluded that all impurity elements are distributed in the volume of the magnesium sulfate crystalline hydrate, with the exception of calcium sulfate, which crystallizes on the surface of magnesium sulfate. Quantitative determination of other impurity elements is not possible due to concentrations below the detection limits of this method.

**Figure 3:**
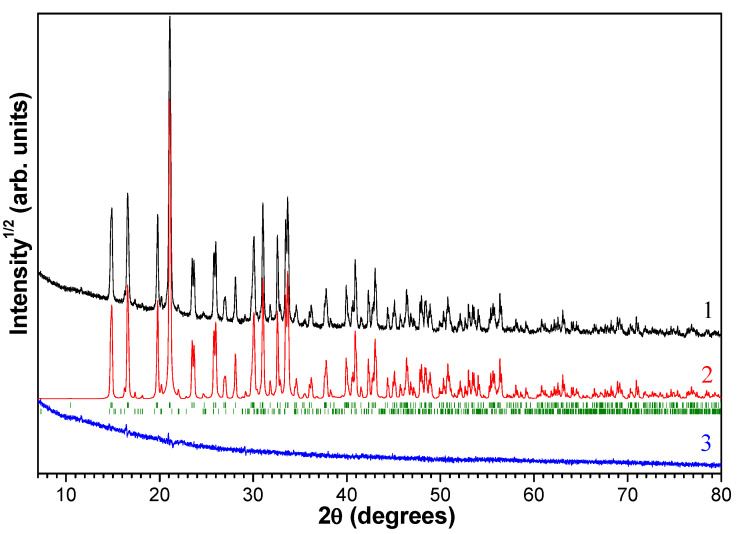
Experimental (**1**), calculated by the DDM method (**2**) and difference (**3**) X-ray patterns of the MgSO_4_·*n*H_2_O sample used as an external standard. The positions of the MgSO_4_∙7H_2_O and MgSO_4_∙6H_2_O peaks are marked with dashes according to the ICDD PDF database.

The structure of the solid desiccant was investigated by means of XRD. The phases present in the samples were identified using the ICDD PDF database [53]. Quantitative determination of phase composition used the method of minimization of derivative difference (DDM) [54], with normalization of the total number of crystalline phases by the X-ray pattern of an external standard. A sample of magnesium sulfate reagent, whose phase composition corresponds to 98.5% of MgSO_4_·7H_2_O and 1.5% MgSO_4_·6H_2_O, was used as an external standard (Figure 3).

**Figure 4:**
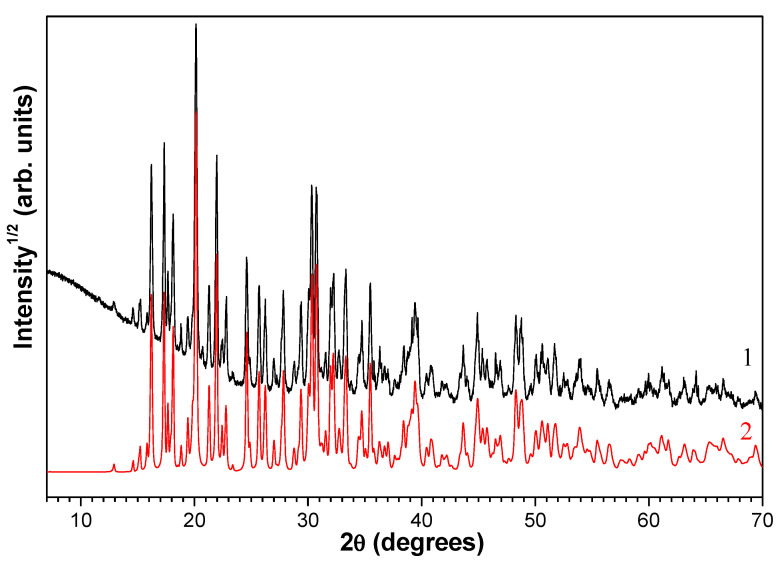
Experimental (**1**) and calculated by the DDM method (**2**) X-ray diffraction patterns of the magnesium sulfate reagent MgSO_4_**·***n*H_2_O −0.2 mm fraction.

Figure 4 shows the X-ray diffraction patterns observed and calculated by the DDM method of the chemical reagent sample—MgSO_4_∙7H_2_O. The sample presents a mixture of crystalline hydrates of 92 wt % MgSO_4_·6H_2_O and 8 wt % MgSO_4_·4H_2_O crystalline hydrates. The observed discrepancy in the number of water molecules in the stoichiometric composition is due to the possibility that crystalline hydrates lose moisture when exposed to air [49].

**Figure 5:**
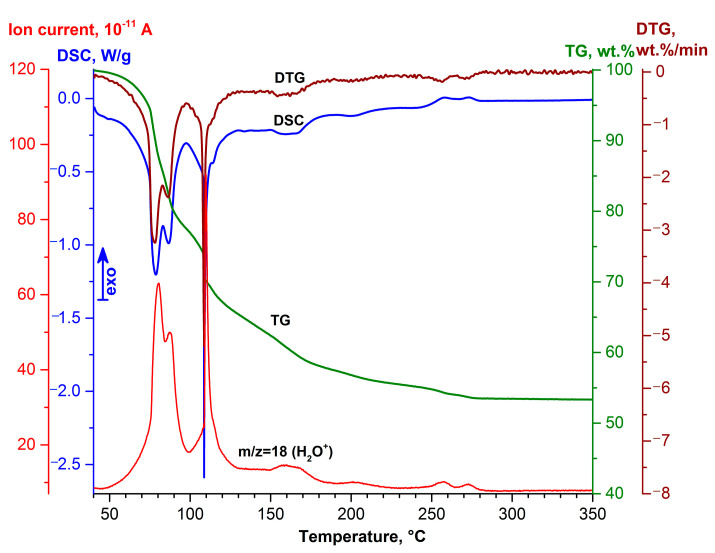
DSC-TG-DTG curves of the thermal transformation process for the magnesium sulfate reagent MgSO_4_**·***n*H_2_O fraction −0.2 mm.

The DSC-TG method allowed us to determine the temperature intervals of dehydration and the amount of water formed during this process for desiccant samples. Analysis of the thermal dissociation results of the magnesium sulfate reagent MgSO_4_·*n*H_2_O −0.2 mm fraction (Figure 5) allows us to identify several peaks in the low temperature region at 79, 87, 109, 165, and 201 °C. Note that the intense DSC peak at 109 °C, accompanied by water release (*m*/*z* = 18), is characteristic of well-crystallized and relatively large crystallites. The mass loss with increasing dehydration temperature increases at 40–350 °C and is 46.67 wt % (Table 1). The final state of MgSO_4_·H_2_O corresponds to a mass loss of 38.69 wt %; the temperature of separation of the last H_2_O molecule is 156.9 °C. The molar ratio of H_2_O/MgSO_4_ in the sample corresponds to 5.8. An insignificant discrepancy between the composition of the crystalline hydrate and XRD data is due to the evaporation of moisture during the isothermal exposure of the sample in the gas mixture flow prior to DSC-TG analysis.

Therefore, thermal dehydration of MgSO_4_·*n*H_2_O with the release of bulk water occurs in the low-temperature region, which allows the desiccant to regenerate at low temperatures. High values of water capacity (Table 1), low regeneration temperature (Figure 5), and positive application experience in agriculture [50,51] determined the choice of magnesium sulfate as an active drying agent in the development of composite desiccant sorbents.

#### 2.1.2. Preparation and Characterization of Microspherical Carriers

The applicability criteria for narrow fractions of cenospheres as functional materials, including carriers and sorbents [39,40,41,42], are compliant with specific requirements for the composition and structure of the glass–crystalline shell [36,37,38]. As is known, the glass–crystalline shell of cenospheres has a complex structure and can be a ring structure with varying degrees of porosity [36,37,39] or a network structure [38,55]. The inner and outer surfaces of the globules have a 30–50 μm thick nanoscale film [36,56] that can be removed with fluorine-containing reagents. As a result, the open porous structure of the glass–crystalline shell and the inner volume of the globules [39,40,41,42] formed by gas inclusions during the formation of cenospheres during coal combustion become accessible.

In this study, microspherical carriers of composite sorbents for contact drying were obtained on the basis of narrow morphologically homogeneous fractions of cenospheres with a predominant content of globules with a porous shell or network structure. Thus, the narrow fraction of cenospheres K −0.5 + 0.25 is fully represented by globules of a ring structure with a porous shell; the narrow fraction of cenospheres R −0.5 + 0.315 contains predominantly foamy particles (79%) with cavities of various sizes (Table 2). Table 2 presents the physical characteristics of these fractions, Table 3 presents their chemical composition, and Table 4 presents their phase composition.

According to chemical analysis, these cenosphere fractions represent a multicomponent system SiO_2_-Al_2_O_3_-Fe_2_O_3_-CaO-MgO-Na_2_O-K_2_O. The contents of the main macrocomponents of SiO_2_ and Al_2_O_3_ are 87 wt % for K −0.5 + 0.25 and 95 wt % for R −0.5 + 0.315 (Table 3). The phase composition of the narrow fraction K −0.5 + 0.25 is 93 wt % of the amorphous glass and crystalline phases; mullite and quartz are present in small amounts. A noticeable difference in the narrow fraction R −0.5 + 0.315 is the higher content of the mullite phase—34 wt %—and the lower content of the glass phase—64 wt % (Table 4).

**Figure 6:**
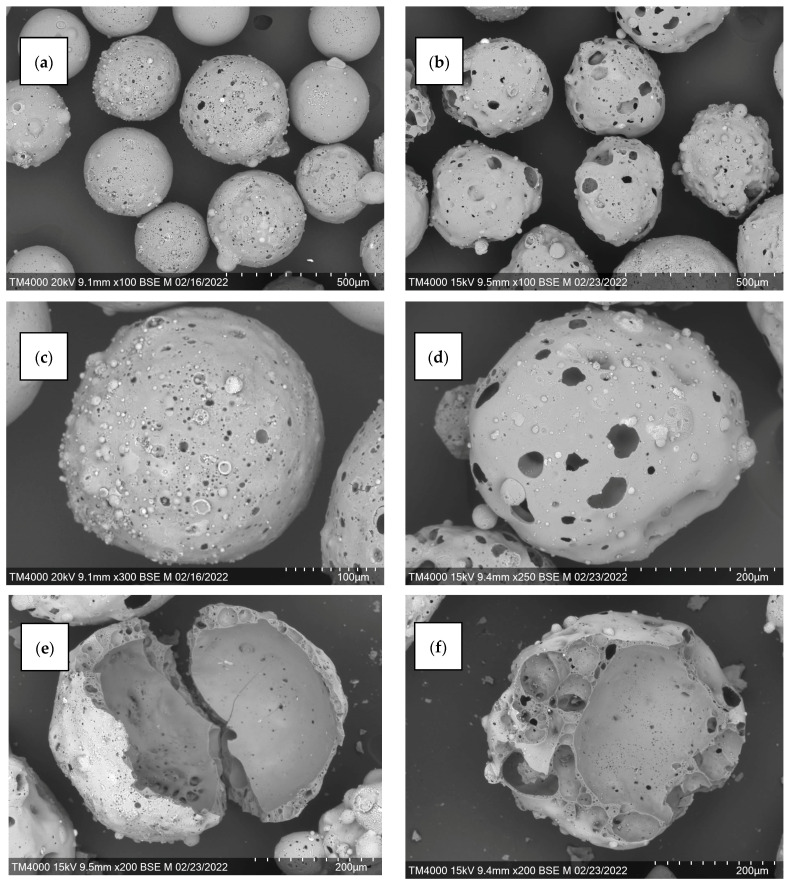

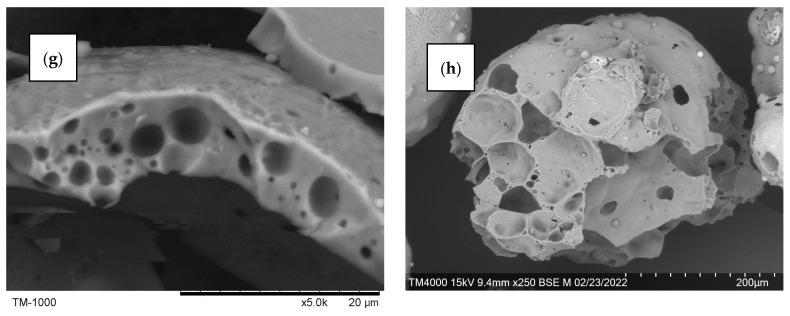
SEM images of narrow fractions of cenospheres and their individual globules after acid etching: (**a**,**c**,**e**,**g**) K −0.5 + 0.25; (**b**,**d**,**f**,**h**) R −0.5 + 0.315. (**a**,**b**) General view; (**c**,**d**) Single globule; (**e**,**f**) Destroyed globule inside; (**g**,**h**) Shell of a destroyed globule.

The preparation of microspherical carriers with through pores and available internal volume to localize the active component involved acid etching of narrow fractions of cenospheres with a reagent based on hydrofluoric acid according to the previously successfully tested method [39,40,41]. The composition of the etching solution was as follows: NH_4_F of the extra pure grade—3.7 g; HCI of the chemically pure grade with a concentration of 12 mol/L—10 mL; and H_2_O (distilled)—up to 100 mL. The volume ratio solid phase/liquid = 1:5; processing time was 15 min. Figure 6 shows SEM images of perforated cenospheres.

#### 2.1.3. Synthesis Procedure and Characterization of Composite Sorbents

The encapsulation of the active component includes the following operations (Figure 1). Perforated cenospheres are pre-vacuumed at a residual pressure of 8–10 kPa, after which a supersaturated water solution of magnesium sulfate (100 g H_2_O + 75 g MgSO_4_·7H_2_O) preheated to a temperature of 80 °C is fed in; the cenosphere/solution ratio = 1:2. At the end of this procedure, the cenospheres, whose inner cavities contain a supersaturated magnesium sulfate solution, are filtered from the excess liquid phase and rinsed with a small amount of distilled water to remove excess magnesium sulfate from the outer surface of the globules. The resulting sorbent is dried in a laboratory oven at a temperature of 105 °C for 90 min. Then, immediately before contact drying, the desiccant is dehydrated at a temperature of 150 °C to a constant weight.

The amount of encapsulated active component (wt %) was calculated according to Formula (1):

(1) $$X\;\;\frac{M_{MS}-M_{0}}{M_{MS}}\times 100,$$

where $M_{MS}$ is the mass of composite sorbent after hosting magnesium sulfate solution in the cenosphere cavities, drying, and dehydration (g) and $M_{0}$ is the mass of the initial perforated cenospheres (g). The amount of active component was indicated in the labeling of microspherical sorbents as MS-X.

**Figure 7:**
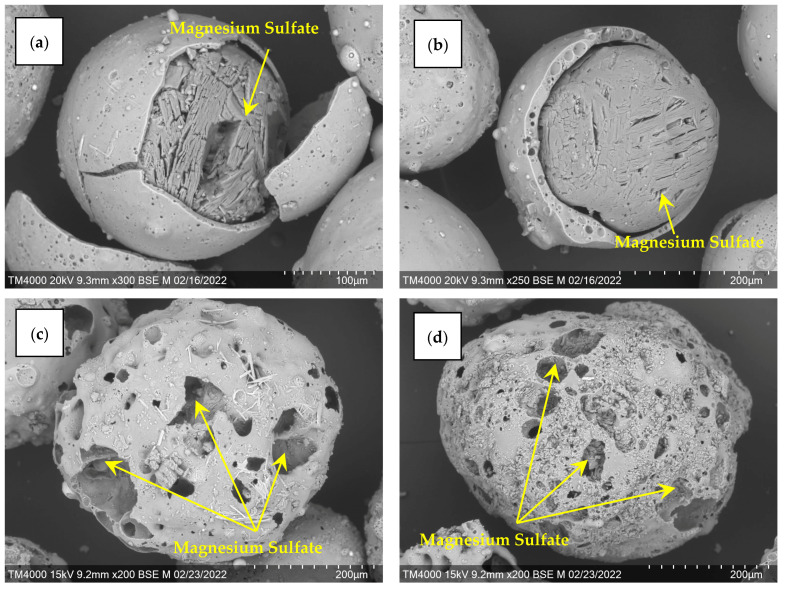
SEM images of composite sorbents: (**a**,**b**) MS-38; (**c**,**d**) MS-26.

For the fraction K −0.5 + 0.25, the amount of active component was 38 wt %, for R −0.5 + 0.315 this value had a lower value of 26 wt %, which is due to the structure and the available internal volume of the porous carrier (Figure 6). The resulting sorbents are labeled MS-38 and MS-26, respectively. SEM images of the microspherical sorbents obtained confirm the localization of the desiccant in the internal cavities of the perforated cenospheres of the ring and network structures (Figure 7).

#### 2.1.4. Texture Characteristics of Microspherical Carriers and Composite Sorbents

The texture characteristics of the samples were characterized by the Brunauer–Emmett–Teller (BET) specific surface area (S_BET_) determined from the absorption branch of the isotherm and by the total pore volume (V_tot_) calculated from the sorbed nitrogen volume at a relative pressure of P/P_0_ ≥ 0.99. The mesopore size distribution was determined by the Barrett–Joyner–Halenda (BJH) method. The micropore volume was assessed using the comparative t-method, with the calculation of the statistical thickness of the adsorbed adsorbate layer according to the Harkins–Jura equation.

The texture characteristics of microspherical carriers and composite sorbents S_BET_, V_tot_ and average pore size are presented in Table 5.

**Figure 8:**
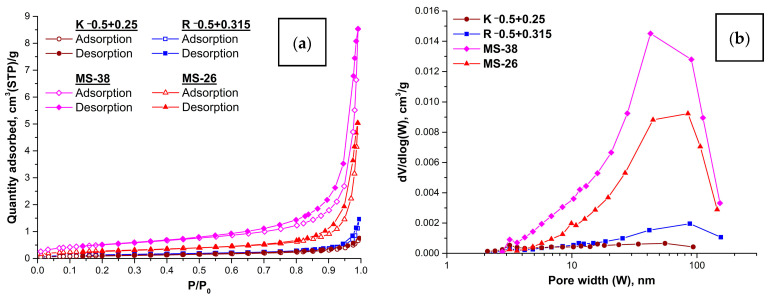
Adsorption–desorption isotherms (**a**) and BJH (from desorption branch) pore size distribution (**b**) for microspherical carriers and composite sorbents.

The isotherms (Figure 8a) of adsorption–desorption of both carriers and composite sorbents, according to the classification of the IUPAC, correspond to Type II, which is typical for meso-/macroporous materials. The presence of a weak H3 hysteresis loop type can indicate the presence of capillary condensation of nitrogen in slit-shaped pores formed by lamellar particles (crystallites) or microcracks.

Microspherical carriers are characterized (Table 5) by a low specific surface area (0.41–0.50 m^2^/g), close to their geometric surface area (0.3–0.4 m^2^/g), as well as a low total pore volume (0.001–0.002 cm^3^/g). The average pore size was 10–14 nm, indicating that mesopores predominate in the material. An assessment of the contribution of micropores using the comparative t-method showed that their volume is close to zero and is within the error of determination.

Analysis of the adsorption–desorption isotherms of microspherical carriers and composite sorbents (Figure 8a) showed that after adding a desiccant the type of isotherm and hysteresis loop remained the same; therefore, at a qualitative level, the texture of the materials did not undergo significant changes. However, the quantitative characteristics of the texture of composite sorbents (Table 5) changed significantly relative to the microspherical carriers:

- The increase in specific surface area was 4.5 times for a sorbent with a ring carrier structure, approximately 2 times for a sorbent with a network carrier structure.
- The total pore volume for a sorbent with a ring carrier structure increased by an order of magnitude, and, in the case of a sorbent with a network carrier structure, a 3-fold increase was observed.
- The average pore size increased approximately 2 times due to an increase in the contribution of meso-/macropores (more than 10–14 nm), which was confirmed by the data of the BJH pore size distribution (Figure 8b), where the main contribution was made by the mesopore size 40–90 nm.

### 2.2. Sorption Drying of Wheat

To determine the water sorption properties of the microspherical composite sorbents, wheat seeds with a moisture content $MC_{0}$ of ~23% were used. Contact drying experiments used an automatic stirring unit that provided uniform container rotation of the mixture. The stirring frequency was 2 rpm. After a specific time had passed from the start of the experiment, the mixture was separated using the sieve method. Measurement of wheat moisture content involved the air–thermal drying method [57,58].

The MS/grain ratio in the contact drying process was 1:2 for MS-38 and 1:1 for MS-26, which, in terms of the active component desiccant/grain mass ratio, is 1:5 and 1:4, respectively. For comparison, the fractions of magnesium sulfate reagent dehydrated at 150 °C were used as powder desiccant samples: coarse −2.0 + 1.0 mm and fine −0.2 mm with desiccant/grain mass ratio 1:2.

The moisture content of wheat at the specific drying time $(MC_{t})\;$with encapsulated microspherical sorbents and powder fractions of desiccant MgSO_4_ is given in Table 6. The data obtained show that in terms of the amount of moisture removed during 5, 30, and 60 min of interaction, composite sorbents are superior to the desiccant fractions even at a low ratio. Then, their sorption activity decreases slightly, which, however, does not prevent them from reaching the required moisture content of wheat after 4 h of contact drying, not exceeding the critical value [43] (Table 6).

Comparison of the moisture sorption properties of the microspherical composite sorbents using the data of Table 6 shows that the maximum value of moisture removal from seeds $(MC_{0}$ − $MC_{t}$) is observed during the first 30 min of contact drying for both sorbents. This value is 3.6% for MS-38 with a ring structure and 3.3% for MS-26 with a network structure.

**Figure 9:**
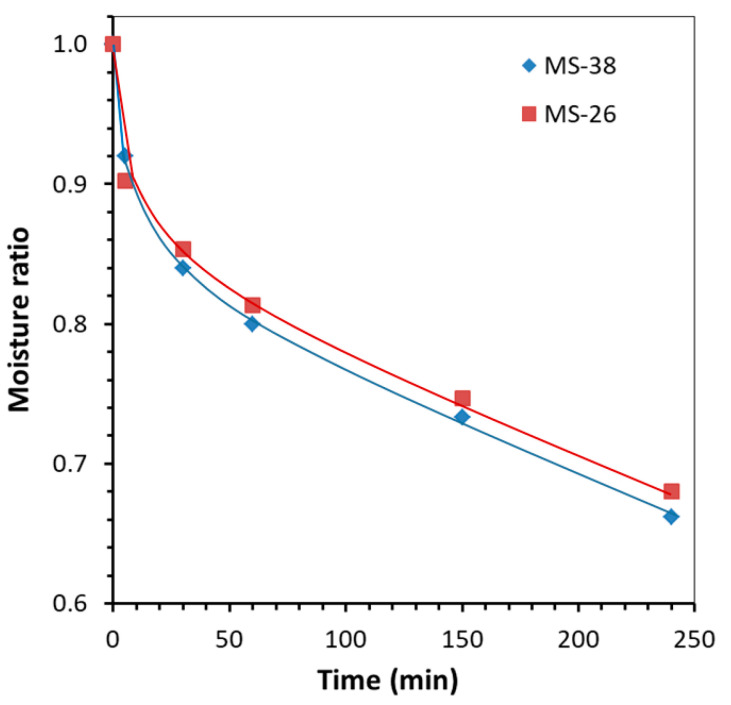
The moisture ratio changes of wheat seeds during contact drying with microspherical composite sorbents.

The change in the moisture ratio of wheat upon contact with the microspherical composite sorbents is shown in Figure 9. The drying curves were plotted according to Equation (2) [43]:

(2) $$MR=\frac{MC_{t}}{MC_{0}}$$

where $MC_{0}$ and $MC_{t}$ are the moisture content on a wet basis (% wb) at the initial stage and at drying time point t, respectively.

Initially, rapid moisture removal is observed for both composite sorbents, but during the drying process the moisture removal rate slows (Figure 9). The decrease in drying rate during this period is associated with the diffusion of moisture within an individual grain of wheat to the surface. It can be seen that the MS-26 sorbent (mass ratio desiccant/grain = 1:4) is somewhat inferior in moisture sorption properties to the sorbent MS-38 (mass ratio desiccant/grain = 1:5), which is due to differences in the structure of the porous carriers (Figure 6) and texture characteristics of the sorbents (Table 5, Figure 8).

**Figure 10:**
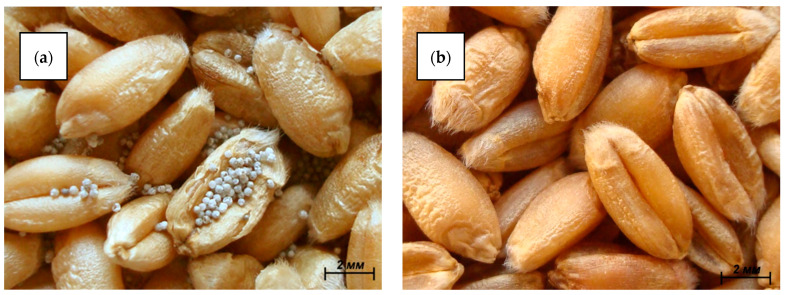
Optical images of wheat with the microspherical composite sorbent MS-38 at the initial stage of contact drying (**a**) and after sieve separation (**b**).

A significant advantage of microspherical composite sorbents is the encapsulation of the active component in the inner volume of the globules, which avoids direct contact with the grain and adherence to the surface. The composite sorbents easily separate at the end of contact drying by the conventional sieve method (Figure 10).

### 2.3. Testing the Cyclicity and Regeneration Ability of Composite Sorbents

After the first drying cycle, the composite sorbents MS-38 and MS-26 were regenerated at 150 °C and reused. The required wheat moisture content was reached in the same period (Table 7) as in the previous cycle (Table 6). This indicates the preservation of the sorption capacity and the possibility of reusing composite sorbents in the “drying”–“regeneration”–“drying” mode.

**Figure 11:**
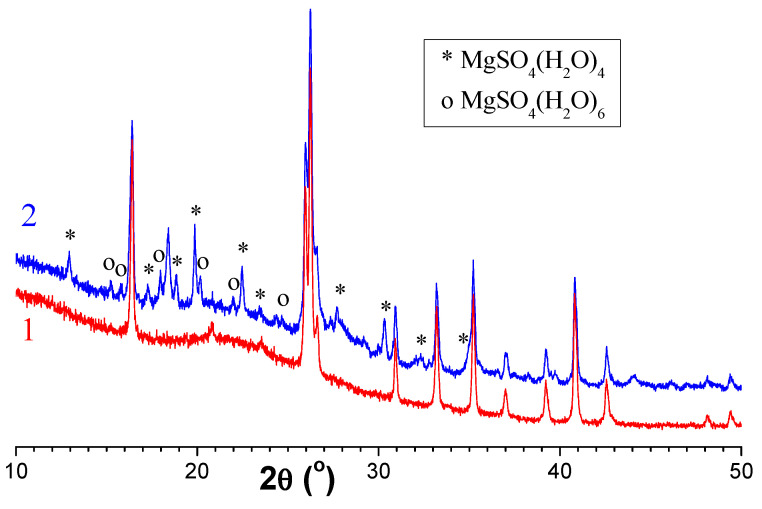
X-ray patterns for the perforated carrier R −0.5 + 0.315 (**1**) and the microspherical sorbent MS-26 (**2**) after two drying cycles with intermediate regeneration. The main peaks of the phases MgSO_4_ (H_2_O)_4_ (ICDD PDF 00-024-0720) and MgSO_4_(H_2_O)_6_ (ICDD PDF 00-024-0719) are marked.

Figure 11 shows the X-ray patterns for the perforated carrier R −0.5 + 0.315 and the microspherical sorbent MS-26, obtained after two drying cycles. As seen, the composite sorbent contains crystalline phases characteristic of the initial cenospheres, mullite and quartz (Table 4) and magnesium sulfate; after contact drying, the formation of MgSO_4_·4H_2_O and MgSO_4_·6H_2_O phases occurs, which confirms the preservation of the active component in the porous carrier of the composite sorbent and its availability as a desiccant.

Reliable fixation of the desiccant in the internal cavities of the globules is due to the structure of the glass–crystalline shell of the cenospheres. This prevents drift of the active component MgSO_4_·*n*H_2_O and its contamination by the dust and organic impurities that accompany crops. Encapsulation of the drying agent makes it possible to maintain the moisture sorption properties of composite sorbent at a consistently high level and to reuse them during post-harvest treatment of grain. Due to the spherical shape of the matrix, it is possible to avoid mechanical damage to the seeds that contributes to the development of microorganisms, which most actively multiply at high moisture content. The determination of wheat seed germination [59,60] after contact drying was 95 ± 2%, which is characteristic of high quality seeds. For comparison, wheat seed germination after open air drying was 94 ± 2%.

## 3. Materials and Methods

### 3.1. Materials

The chemical reagent magnesium sulfate 7-hydrate of “pure” grade, MgSO_4_∙7H_2_O, GOST 4523-77 (JSC Chemical Plant, Mendeleevsk, Russia) was used as a solid desiccant.

Fly ash cenosphere concentrates obtained from the pulverized combustion of coal from the Kuznetsk and Ekibastuz basins were used as raw materials to obtain narrow morphologically homogeneous fractions of cenospheres. The narrow cenosphere fractions were separated according to a technological scheme [36,37,38] including stages of aerodynamic classification, magnetic separation, granulometric classification, and hydrostatic separation from broken globules. As a result, a non-magnetic narrow fraction with a size of −0.5 + 0.25 mm (series K) was separated from the fly ash cenosphere concentrate after combustion of Kuznetsky coal at Novosibirskaya TPP-5 (Siberian Generating Company LLC, Novosibirsk, Russia). From the concentrate of the cenospheres of fly ash after combustion of Ekibastuz coal at Reftinskaya TTP (JSC Kuzbassenergo, Kemerovo, Russia) a non-magnetic fraction with a size of −0.5 + 0.315 mm was obtained (series R).

Wheat seeds (*Triticum aestivum* L.) used in this study belonged to the Novosibirskaya 41 grade variety produced in the East Siberian region (EPF “Mikhailovskoye”, FRC KSC SB RAS, Krasnoyarsk, Russia) and harvested in 2022.

### 3.2. Characterization Techniques

The moisture content of wheat was measured using the air–thermal drying method at a temperature of 130 ± 2 °C according to GOST 13586.5-2015 [57] and the international standard ISO 712:2009 [58].

Wheat seed germination was evaluated following a standard test according to GOST 12038–84 [59] and the international rules for seed testing [60]. The test was carried out using four replicates, each comprising 100 seeds, using the filter paper method at a temperature of 20 °C. Seed germination was assessed on the seventh day of the experiment after seed was planted.

The SEM-EDS study of solid desiccant used a TM-4000 scanning electron microscope (High Technologies Corporation, Hitachi, Tokyo, Japan) equipped with a Quantax 70 microanalysis system and a Bruker XFlash 430H energy-dispersive X-ray spectrometer (Bruker Corporation, Billerica, MA, USA) at a magnification of ×50–1000 and an acceleration voltage of 20 kV. Powder samples were applied to a double-coated conductive carbon adhesive tape (Ted Pella Inc., Altadena, CA, USA) attached to a flat substrate (1–3 mm thick, 30 mm in diameter) fabricated from Duopur poly(methyl methacrylate) resin (Adler, Schwaz, Austria).

X-ray studies of the desiccant used a PANalytical X’Pert PRO diffractometer (PANalytical, Almelo, The Netherlands) equipped with a PIXcel detector and a graphite monochromator on CuKα radiation. The packaging of desiccant fractions in a cuvette under a thin polymer film insulated with vacuum grease without grinding prevented the exchange of moisture with the atmosphere during imaging. The scanning of the X-ray patterns was at ~25 °C in the range of diffraction angles 5° ≤ 2θ ≤ 80° with a step of 0.013°. Each sample was scanned 2–3 times within 1 h to control for possible changes in the material during imaging. Subsequent comparison of repeated scans did not reveal any noticeable changes in the X-ray patterns, indicating reliable isolation of the material from the atmosphere.

Simultaneous thermal analysis (DSC–TG) of solid desiccant was performed in a dynamic gas mixture of 20% O_2_ + 80% Ar with a total flow of 50 sccm, with simultaneous registration of mass changes and heat flow on a Jupiter STA 449C synchronous thermal analysis unit with an Aëolos QMS 403C mass spectral (MS) analyzer (Netzsch, Selb, Germany). Measurements were performed in Pt-Rh crucibles with perforated lids at a linear temperature rate of 2.5 °C/min within 40–350 °C with a sample weight of 11 ± 1 mg. Before heating, isothermal exposure occurred under the flow of the gas mixture for 5 min at 40 °C. The DSC sensor was calibrated for heat flow by measuring the heat capacity of the sapphire disk according to DIN 51007:1994-06 [61], Thermal analysis; differential thermal analysis; principles. Primary thermo-analytical data processing was performed using the licensed NETZSCH Proteus (ver. 4.8.4) software package.

The texture characteristics of the samples were determined from the nitrogen adsorption and desorption isotherms measured at −196 °C under relative pressures P/P_0_ from 0.005 to 0.995 using the ASAP 2020 adsorption automatic analyzer Micromeritics (Micromeritics Instrument Corporation, Norcross, GA, USA). Before measurements, samples were degassed at a temperature of 200 °C for 24 h under vacuum.

For narrow fractions of the cenospheres, the following physicochemical characteristics were determined: bulk density, chemical and phase composition, average diameter of the globules, apparent thickness of glass–crystalline shells, and the content of the globules related in structure to a particular morphological type. The methods to determine these parameters are described in detail in [36,37,38].

Bulk density was measured on an automated Autotap density analyzer (Quantachrome Instruments, Boynton Beach, FL, USA).

Chemical composition was determined using chemical analysis according to GOST 5382-2019 [62], which specifies the methods for component identification [36].

Phase composition was determined using quantitative X-ray powder diffraction analysis with the full-profile Rietveld method and the derivative difference minimization according to procedure [37].

The average diameter of the globules, the apparent thickness of the glass–crystalline shells, and the contents of globules of different morphological types were studied using an Axioscop Imager D1 optical microscope equipped with an AxioCam MRc5 color digital camera (Zeiss, Carl-Zeiss-Stiftung, Oberkochen, Germany) [38].

The study of the structure of the cenosphere shells used TM-4000 scanning electron microscopes (High Technologies Corporation, Hitachi, Tokyo, Japan).

## 4. Conclusions

New microspherical composite sorbents have been developed for energy-saving drying technology of grain and seeds of crops using magnesium sulfate as the active moisture sorption component. To localize the active component, porous carriers with accessible internal volumes and perforated glass–crystalline shells obtained by acid etching of narrow fractions of cenospheres were used. Studies of the moisture sorption properties of composite sorbents showed that after 4 h of contact drying the moisture content of wheat seed was 14.9–15.5 wt %. Wheat seed germination after contact drying was 95 ± 2%. The advantage of composite sorbents is the encapsulation of the active component in the inner volume of globules, which prevents entrainment, contamination, and adhesion to the surface. The microspherical composite sorbents obtained retained stable sorption capacity at a high level over several drying cycles. The study results can serve to develop non-thermal and sustainable technologies for the contact drying of thermolabile materials that do not tolerate thermal effects or else lose valuable properties when heated.

## Figures and Tables

**Table 1:** Mass loss (∆m) and water capacity in the temperature range 40–350 °C.

Parameter	Temperature Range (°C)					
	40–100	40–150	40–200	40–250	40–300	40–350
∆m (wt %)	23.04	37.61	43.19	45.21	46.54	46.67
Water capacity (mg/g) *	432	705	810	848	873	875

* per gram of anhydrous substance.

**Table 2:** Physical characteristics and content of cenospheres with specific morphology.

Fraction	Physical Characteristics			Content of Cenospheres (%)	
	Bulk Density (g/cm^3^)	Average Diameter (μm)	Apparent Thickness of Shell (μm)	Single-Ring Structure with Porous Shell	Network Structure
K −0.5 + 0.25	0.38	280	11	100	0
R −0.5 + 0.315	0.40	383	19	21	79

**Table 3:** Chemical composition of narrow fractions of cenospheres (wt %).

Fraction	LOI	SiO_2_	Al_2_O_3_	Fe_2_O_3_	CaO	MgO	Na_2_O	K_2_O
K −0.5 + 0.25	0.64	66.58	20.54	3.02	2.30	2.30	1.35	3.25
R −0.5 + 0.315	0.80	54.82	39.80	1.10	1.54	1.09	0.30	0.21

**Table 4:** Phase composition of narrow fractions of cenospheres (wt %).

Fraction	Glass Phase	Mullite	Quartz	Calcite
K −0.5 + 0.25	92.7	1.2	5.5	0.6
R −0.5 + 0.315	64.1	34.0	1.7	0.2

**Table 5:** Textural characteristics of microspherical carriers and composite sorbents.

Fraction	S_BET_ (m^2^/g)	V_tot_ (cm^3^/g)	Average Pore Size (nm)
K −0.5 + 0.25	0.41	0.001	10.0
R −0.5 + 0.315	0.50	0.002	14.0
MS-38	1.87	0.010	21.9
MS-26	1.01	0.006	25.4

**Table 6:** Moisture content (wt %) of wheat seeds during contact drying.

Sample	Desiccant/Grain Mass Ratio	Time (min)					
		0	5	30	60	150	240
MS-38	1:5	22.5	20.7	18.9	18.0	16.5	14.9
MS-26	1:4	22.5	20.3	19.2	18.3	16.8	15.3
MgSO_4_ −2.0 + 1.0 mm	1:2	21.4	20.6	19.2	18.5	15.8	14.9
MgSO_4_ −0.2 mm	1:2	22.6	21.0	19.5	18.4	16.0	14.4

**Table 7:** Moisture content (wt %) of wheat seeds during contact drying with a microspherical composite sorbent after regeneration in the second cycle.

Sample	Desiccant/Grain Mass Ratio	Time (min)					
		0	5	30	60	150	240
MS-38 (2nd cycle)	1:5	22.5	20.6	18.8	18.2	16.6	15.1
MS-26 (2nd cycle)	1:4	22.5	20.4	19.4	18.0	16.8	15.5

## Data Availability

The data presented in this study are available on request from the corresponding author.
